# Causes and Potential Solutions of Molar Buccal Inclination in Maxillary Skeletal Expander: A Retrospective CBCT Analysis

**DOI:** 10.1155/ijod/6975568

**Published:** 2025-11-21

**Authors:** Hongkun Sun, Nan Wei, Chiyuen Cheung, Yi Feng, Yiting Shao, Hong Ai, Shaoqing Tu, Zheng Chen

**Affiliations:** Department of Stomatology, The Third Affiliated Hospital of Sun Yat-Sen University, 600#, Tianhe Road, Guangzhou, Guangdong, China

**Keywords:** appliance, cone-beam computed tomography, orthodontic treatment, palatal expansion technique

## Abstract

**Objective:**

This study aimed to investigate the causes of molar buccal inclination following maxillary skeletal expander (MSE) treatment and explore potential solutions by analyzing MSE appliance design and patient-specific factors to mitigate this inclination.

**Materials and Methods:**

Cone-beam computed tomography (CBCT) data from 120 patients with maxillary transverse deficiency (MTD) were analyzed using Materialise's Interactive Medical Image Control System (MIMICS) software. The buccal inclination of the zygomaticomaxillary complex (ZMC), alveolar bone, and molars was measured using angular analysis to identify the contributing factors to molar buccal inclination.

**Results:**

Significant changes were observed in the frontozygomatic angle (FZA), frontoalveolar angle (FAA), frontodental angle (FDA), ZMC angle (ZMCA), maxillary first molar angle (MFMA), and maxillary second molar angle (MSMA) (*p* ≤ 0.001, *p* < 0.05). ΔFZA was less than ΔFDA (*p*=0.019, *p* < 0.05), while no significant difference was found between ΔZMCA and ΔMSMA (*p*=0.390, *p* > 0.05). However, a significant difference was noted between ΔZMCA and ΔMFMA (*p* ≤ 0.001, *p* < 0.05). The ratio of ΔZMCA/ΔMFMA was higher in the age ≤18 group (0.712 ± 0.044) compared to the age >18 group (0.511 ± 0.048) (*p* ≤ 0.001, *p* < 0.05).

**Conclusions:**

During MSE expansion, the buccal inclination of the molars will still occur, primarily due to the buccal expansion force transmitted to the molars by the bracket and the band. Additionally, this inclination is partly influenced by the left and right ZMC rotating buccally in the coronal plane.

## 1. Introduction

Maxillary transverse deficiency (MTD) is a skeletal discrepancy characterized by reduced width of the maxillary basal bone, disrupts the transverse harmony between the maxillary and mandibular skeletal bases [1]. Epidemiological studies reported that MTD prevalence rates of 8.0%–23.3% in primary and mixed dentition populations and 9.4% in permanent dentition cohorts [2–6]. Clinically, this condition presents with a constricted maxillary arch, posterior crossbite (unilateral or bilateral), dental crowding, and an elevated palatal vault [7]. Untreated MTD may progess to functional mandibular deviation, musculoskeletal dysfunction, and impaired oral and maxillofacial function, as well as compromised facial esthetics [8]. Severe cases are associated with nasal stenosis, ventilation dysfunction, and obstructive sleep apnea syndrome (OSAS), substantialy compromising patients' quality of life and overall health [9]. Therefore, early diagnosis and intervention are critical to reestablish maxillomandibular transverse balance and optimize maxillary growth potential [10].

For young adolescents, rapid palatal expansion (RPE) is an effective treatment for MTD [11]. The RPE-hyrax appliance transmits orthopedic forces bilaterally through anchored first molars and premolars to the mid-palatalsuture, facilitating skeletal expansion in younger individuals with patent sutures [12]. Treatment efficacy diminishes significantly postadolescence, as progressive fusion of the mid-palatal suture and circummaxillary articulations increases resistance to skeletal expansion [13–15]. In late-adolescents and adults populations, RPE predominantly induces dentoalveolar effects, including molar buccal tipping and alveolar remoding, with limited skeletal contribution [16]. Research shows that in children, approximately 50% of RPE-induced expansion is skeletal, while the remaining 50% is dentoalveolar. In adolescents, this ratio shifts to 35% skeletal and 65% dental [17, 18]. Additionally, excessive orthopedic forces during RPE may precipitate adverse outcomes such as alveolar bone loss, fenestration, and dehiscence of anchored teeth [19].

The advent of temporary anchorage devices (TADs) has revolutionized MTD treatment, enabling skeletal expansion with reduced dentoalveolar side effects. The maxillary skeletal expander (MSE), a type of microscrew-assisted RPE (MARPE), developed by Dr. Moon at the University of California, Los Angeles (UCLA), has gained prominence as a nonsurgical solution for adult maxillary width deficiency [20]. While MSE achieves greater skeletal effects than conventional RPE [21], a consistent complication is buccal crown inclination of the maxillary first molars [22–24].

Post-MSE buccal molar inclination poses multifactorial risks, including occlusal disharmony, alveolar bone thinning, and root displacement. In brief, molar buccal inclination usually accompany with molar extrusion, which may alter temporomandibular joint dynamics by enlarging the superior and posterior joint spaces [25, 26]. Additionally, the buccal alveolar plates often exhibit cortical thinning, fenestration, or dehiscence due to uneven force distribution [24, 27, 28]. Furthermore, the synergistic effects of buccal tipping and high-magnitude expansion force increases root resorption risks [29]. Despite clinical recognition of these sequelae, the etiological mechanisms driving post-MSE molar tipping remain underexplored. This study employs cone-beam computed tomography (CBCT) to systematically analyze causative factors, including device design and patient-specific variables, with the objective of informing clinical strategies to mitigate this complication.

## 2. Materials and Methods

This research adhered to the principles of the Declaration of Helsinki and received ethical approval from the Third Affiliated Hospital of Sun Yat sen University Ethics Committee (Protocol number 2021030). This retrospective study included 120 patients (51 males, 69 females) with maxillary transverse deficiency (MTD) who underwent MSE expansion treatment at the Stomatology Center of the Third Affiliated Hospital of Sun Yat sen University between 2018 and 2024. The mean age of the participants was 17.25 ± 1.79. All patients provided written informed consent. The inclusion criteria were as follows: (1) a maxilla-mandible width difference of <5 mm, as determined by the University of Pennsylvania CBCT Analysis, indicating MTD [30]; (2) no history of maxillary dilatation or orthognathic surgery; (3) no cleft lip, cleft palate, and other serious dental deformities.

CBCT scans were performed using the New Tom imaging system (Cefla s.c., Italy). The scanning parameters were as follows: scanning time of 18–26 s, voxel size of 0.075 μm, slice thickness of 0.3 mm, tube current of 1 mA, and field of view of 4000 (L) × 1300 (W) × 2600 (H) mm. All scans were conducted by the same technician in the radiology department of the Third Affiliated Hospital of Sun Yat sen University. The raw CBCT data were exported in DICOM format and reconstructed three-dimensionally using Materialise's Interactive Medical Image Control System ([MIMICS] version 21.0; Materialise, Leuven, Belgium). During image acquisition, patients were seated upright with the chin stabilized in a natural head position. The Frankfort horizontal plane was maintained parallel to the ground, with the teeth in maximum intercuspation. Lips were relaxed in passive closure, and subjects breathed quietly while maintaining static posture of the perioral musculature and tongue. Swallowing or other voluntary movements were prohibited during scanning.

**Figure 1:**
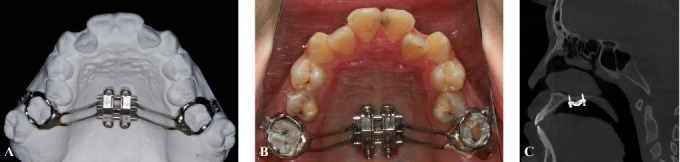
The use of MSE. (A) The structure of MSE. (B) Placement of MSE. (C) The sagittal plane in CBCT and mini-implants passed through the double cortex of the maxilla.

Prior to each patient undergoing expansion therapy, the implant position is strictly determined based on pretreatment CBCT images, and an appropriately sized implant is selected according to the measured thickness of the double cortex of the maxilla to ensure complete adaptation between the implant and the bilateral cortical bone. Upon completion of implantation, a lateral cephalometric radiograph is taken to verify the implantation effect. Each patient was treated with MSE type II (BioMaterials Korea Inc., Seoul, Korea), which was designed the position on maxilla by digital dental model. The appliance consisted of two stainless steel brackets welded to an alloy cast band, which was bonded to the maxillary first molars. Under local infiltration anesthesia, four mini-implants (length: 11 or 13 mm; diameter: 1.5 mm; Mplant-U3-11, BioMaterials Korea Inc., Seoul, Korea) were inserted through the guide grooves of the MSE type II, ensuring bicortical engagement. The mini-implants were positioned 3 mm from the mid-palatal suture and passed through the bilayer cortical bone of the maxilla (Figure 1). Expansion protocols varied by age: (1) patients ≤18 years old turned the appliance twice daily and (2) patients aged >18 years turned the appliance 4–6 times daily. Expansion continued for approximately 2 weeks until the lingual cusp of the maxillary first molar occluded with the buccal cusp of the mandibular first molar. CBCT scans were obtained before (T1) and after (T2) expansion.

**Figure 2:**
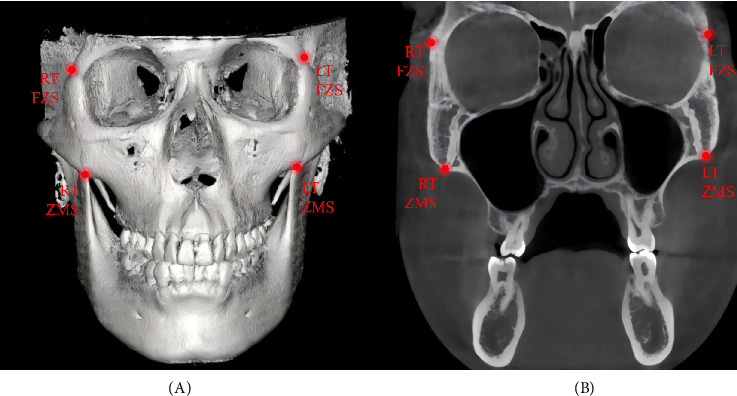
(A) 3D reconstruction with the coronal zygomatic section passing through the right and left frontozygomatic sutures (FZS) and zygomaticomaxillary sutures (ZMS). (B) Corresponding coronal ssection in A.

**Figure 3:**
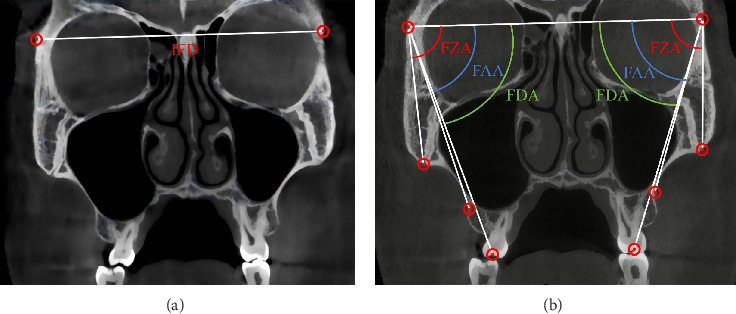
Determine the pivot point and measure angles based on it using (A) reference lines to determine rotational fulcrum, interfrontal distance (IFD), (B) frontozygomatic angle (FZA), frontoalveolar angle (FAA), and frontodental angle (FDA).

The CBCT analysis method adopted in this study was based on angular measurements as described by Paredes et al. [31]. Paredes et al. identified the pivot point for zygomaticomaxillary complex (ZMC) rotation near the external surface of the frontozygomatic suture (FZS), as sutures represent the weakest points in midfacial structures during expansion [32]. The pivot was determined by identifying the point of maximum interfrontal distance, which remained unchanged before and after MSE treatment. In current study, the same method was used to locate the ZMC rotation pivot [31]. The coronal zygomatic plane was defined as the section passing through the uppermost point of the FZS and the lowermost point of the zygomaticomaxillary suture (ZCS) (Figures 2 and 3A). The maximum interfrontal distances before (d1) and after (d2) treatment were measured. If |d1 – d2| ≤ 0.1 mm, the farthest interfrontal distance point was considered the ZMC pivot. If |d1–d2| > 0.1 mm, the pivot was adjusted upward until the condition was met.

**Figure 4:**
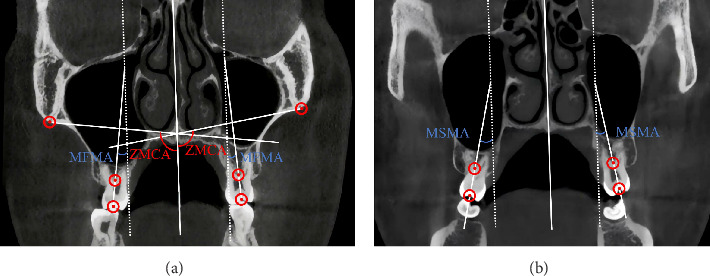
Angle measurement: (A) maxillary first molar angle (MFMA), zygomaticomaxillary complex angle (ZMCA). (B) Maxillary second molar angle (MSMA). The dashed line is the translation line of the center line.

The following angular measurements were performed from the pivot point (Figures 3B and 4) as shown:

1. Frontozygomatic angle (FZA): The angle between the interfrontal line and the line extending from the pivot to the most external point of the ZCS.
2. Frontoalveolar angle (FAA): The angle between the interfrontal line and the line extending from the pivot to the alveolar bone surface at the level of the distobuccal root tip of the maxillary first molars.
3. Frontodental angle (FDA): The angle between the interfrontal line and the line extending from the pivot to the occlusal point at the central groove of the maxillary first molar.
4. ZMC angle (ZMCA): The angle between the facial midline and the line connecting the ZCS and the junction of the cortical bone forming the nasal cavity and the maxillary sinus floor.
5. Maxillary first molar angle (MFMA) and maxillary second molar angle (MSMA): The angle between the line connecting the central fossa of the molar crown and the root furcation and the facial midline. MSMA was not measured if the maxillary second molar had not established an occlusal relationship.

Data were collected with clinicians from the department of Stomatology, The Third Affiliated Hospital of Sun Yat-sen University, under the direct supervision and quality control of the first and corresponding authors. A standardized protocol was implemented, and all personnel were trained to ensure consistency. Clinical measurements were performed in triplicate, with averages used for analysis. Subsequent data cleaning, analysis, and interpretation were conducted exclusively by the authors per the formal data usage agreement for this ethically approved study.

The sample size was estimated using PASS 2021 software (version 21.0.3; NCSS) based on preliminary CBCT data from 20 patients. An *F*-test indicated that a minimum of 114 samples were required; thus, data from 120 patients were collected. Statistical analyses were performed using SPSS software (version 21.0; IBM). Measurements of d1, d2, FZA, FAA, FDA, ZMCA, MFMA, and MSMA at T1 and T2 were analyzed. The Δ was used to denote the change in angle between T1 and T2, calculated as: (Δ) = (T1 measured value) − (T2 measured value). The Kolmogorov–Smirnov test confirmed the normal distribution of ΔFZA, ΔFAA, ΔFDA, ΔZMCA, ΔMFMA, and ΔMSMA. A one-way analysis of variance (ANOVA) was used to assess differences among ΔFZA, ΔFAA, and ΔFDA, followed by pairwise comparisons using the least significant difference (LSD) method. Independent samples *t*-tests were used to compare ΔZMCA with ΔMFMA and ΔMSMA, as well as to evaluate differences between patients ≤18 years old and those >18 years old. A *p*-value <0.05 was considered statistically significant.

## 3. Result

Since |d1–d2| ≤ 0.1 mm, the maximum interfrontal distance remained unchanged before and after treatment. Consequently, the farthest interfrontal distance points were identified as the pivot of the ZMC. The measurements of FZA, FAA, FDA, ZMCA, MFMA, and MSMA are presented in Table 1. All measurements followed a normal distribution (*p*=0.25, *p* > 0.1), and the differences between T2 and T1 were statistically significant (*p* ≤ 0.001, *p* < 0.05).

The mean changes in the measured angles were as follows: ΔFZA (1.197 ± 0.227), ΔFAA (1.195 ± 0.282), ΔFDA (1.615 ± 0.233), ΔZMCA (2.036 ± 0.403), ΔMFMA (4.315 ± 0.786), and ΔMSMA (2.313 ± 0.399). ANOVA confirmed homogeneity of variance (*p*=0.206, *p* > 0.05) and revealed statistically significant differences among ΔFZA, ΔFAA, and ΔFDA (*p*=0.023, *p* < 0.05). Pairwise comparisons using the LSD method showed a statistically significant difference between ΔFZA and ΔFDA (*p*=0.019, *p* < 0.05). No significant difference was observed between ΔZMCA and ΔMSMA (*p*=0.390, *p* > 0.05), whereas the difference between ΔZMCA and ΔMFMA was statistically significant (*p* ≤ 0.001, *p* < 0.05), as detailed in Table 2. The average ratio of ΔZMCA to ΔMFMA was 0.5718 ± 0.0849.

**Figure 5:**
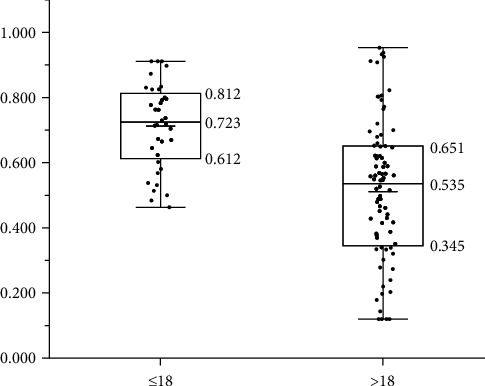
The proportion distribution of buccal inclination of zygomaticomaxillary complex in maxillary first molar in different age groups.

Of the 120 patients, 36 were ≤18 years old and 84 were >18 years old. No significant difference was found in ΔMFMA between the ≤18 group (6.728 ± 2.786) and the >18 group (8.900 ± 2.744), and there is no significant difference between the male and female (*p*=0.272, *p* > 0.05). However, the ratio of ΔZMCA to ΔMFMA differed significantly between the ≤18 group (0.712 ± 0.044) and the >18 group (0.511 ± 0.048), as shown in Table 3. The distribution of these ratios is illustrated in Figure 5.

## 4. Discussion

The MSE has become a cornerstone in skeletal maxillary expansion due to its predictable outcomes and reduced dental side effects relative to traditional RPE [33]. Studies demonstrate MSE achieves 84%–87% success rate in mid-palatal suture separation, producing a more parallel expansion pattern (90% anteroposterior ratio) compared to RPE's anteriordominant opening [20, 34]. Additionally, MSE induces favorable three-dimensional craniofacial remodeling, including increased nasal cavity and nasopharynx volume, and periorbital skeleton widening [35, 36]. While its force distribution through bicortically anchored mini-implants minimizes molar displacement [37, 38], challenges persist in late adolescents and adults, notably molar buccal inclination and palatal cusp drop [24, 31, 39].

This study hypothesized that the molar buccal inclination observed in previous studies is not solely caused by the expansion force, but also results from the molars being part of the ZMC structure, which undergoes rotation during maxillary expansion. Additionally, the buccal inclination of the maxillary first molar is strongly associated with the buccal force exerted by the band, and this inclination can be reduced after the band's removal.

The statistically significant changes in ΔFZA, ΔFAA, and ΔFDA indicate that the ZMC, alveolar bone of the maxillary first molar, and the maxillary first molar itself all rotate buccally around a pivot located near the external surface of the FZS. This finding aligns with previous studies [40, 41]. Furthermore, the significant difference between ΔFZA and ΔFDA suggests that while the maxillary first molar rotates buccally with the ZMC, it also exhibits additional buccal rotation relative to the ZMC. Thus, the total molar buccal inclination is the sum of ZMC rotation and molar rotation. On average, ΔZMCA accounted for 57.18% of ΔMFMA, indicating that 57.18% of the molar inclination is due to ZMC rotation, while 42.82% results from molar rotation.

**Figure 6:**
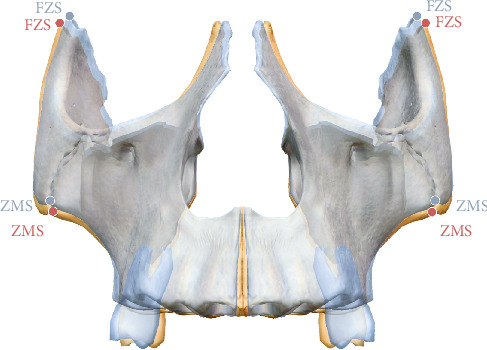
Illustration for the buccal inclination of zygomaxillary complex and maxillary first molar: before treatment is in red and after treatment is in blue, frontozygomaticsutures (FZS) and zygomaticomaxillary sutures (ZMS).

The maxillary second molar, which lacks direct MSE force application, showed no significant difference between ΔZMCA and ΔMSMA. Compared with the maxillary first molar, buccal inclination was almost entirely due to the buccal rotation of the ZMC. So far, this study can basically verify the hypothesis proposed. When MSE is used in the standard procedure, the buccal inclination of the molars will still occur, primarily due to the buccal expansion force transmitted to the molars by the bracket and the band, and partly due to the left and right ZMC buccal rotating in the coronal plane, and as the structure of the ZMC, the molar exhibits the spatial effect (Figure 6). However, this study assessed molar inclination immediately after expansion (T2) but did not evaluate long-term stability or potential relapse. Further follow-up studies are needed to determine whether the observed buccal inclination persists or self-corrects over time.

To optimize clinical outcomes, in addition to achieving 6–8 months of maintenance, three strategic modifications are proposed:

Age considerations: Previous CBCT studies have suggested that 18 years of age is the threshold for the midpalatal suture maturity [42, 43]. Therefore, this study analyzed whether there are differences in buccal inclination of maxillary first molar and the ratio of buccal inclination of ZMC to maxillary first molar inclination in ≤18 and >18 group. The average buccal inclination of maxillary first molar in ≤18 group was lower than that in >18 group, although the difference was not statistically significant, the ratio of ZMC buccal inclination to maxillary first molar buccal inclination in ≤18 group was greater than that in >18 group, and molar inclination was more caused by ZMC rotation. For older juveniles, MSE is conducive to maintaining the effect of expansion [38], so MSE expansion in older juveniles may further reduce the buccal inclination of the maxillary first molar. However, there is a lack of studies on molar inclination in older juveniles receiving RPE and MSE expansion, which is the direction of our research later.

Mini-implant placement: This study found that the ZMC developed a buccal inclination after MSE treatment. Studies have found that all four mini-implants passing through the double cortex of the maxilla can obtain a more parallel transverse expansion in the coronal midpalatal suture compared with only passing through the single cortex of the maxilla [44]. Therefore, bicortical mini-implant can reduce the buccal inclination of the ZMC and thus reduce the buccal inclination of the molar. In addition, the region on both sides of midpalatal suture around the first molar is preferred for mini-implants placement, because the thickness of the bone in this area is perpendicular to the direction of expansion. In the case of hard palate bone support, the posterior micro-implant should be placed as far back as possible to resist the resistance of posterior maxilla, zygomatic column, pterygopalatine suture, and other parts, so as to ensure the parallel expansion of midpalatal suture as much as possible. Meanwhile, through a comprehensive evaluation of each patient's CBCT images and three-dimensional maxillary models, precise anatomical localization is ensured in avoiding the greater palatine foramen, neurovascular bundles, and other critical structures, so as to prevent iatrogenic injuries.

**Figure 7:**
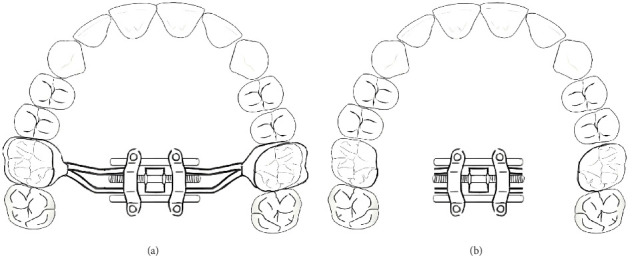
Schematic drawing for attempt: (A) Placement of MSE. (B) After the implantation of the mini-implants, remove the stainless steel brackets and the maxillary first molar band.

The band on molar: The MSE was found to be a MSE, but it still caused the molar with the band to buccal inclination relative to the ZMC. It has been found that although MSE can reduce the molar buccal inclination compared with RME, there is still a large stress on the palatal and palatal root furcation of the maxillary first molar, and a large tension on the palatal root apex of the first molar [37], leading to the molar inclination and the alveolar bone inclination [45]. Due to the stress of the MSE arm in the area around the molar [46], after the implantation of the mini-implants, if the maxillary first molar has no serious crossbite requiring treatment and there is a risk of fenestration or dehiscence of the alveolar bone, the stainless steel brackets and the maxillary first molar band can be tried to remove to relieve the force on the maxillary first molar during the MSE expansion, thereby reducing the buccal inclination of the maxillary first molar (Figure 7).

The mini-implant-assisted expander, particularly MSE, has expanded treatment options for MTD. By optimizing patient selection, mini-implant placement, and appliance using, clinicians can minimize adverse effects such as molar buccal inclination and enhance clinical outcomes. Further research is needed to refine these strategies and improve the efficacy of MSE in diverse patient populations.

Furthermore, this study obtained the research data by taking CBCT images. CBCT was essential to obtain accurate, three-dimensional data on both dental changes and skeletal effects, which are fundamental to the aims of this study. A rigorous risk–benefit analysis was performed. All participants received detailed radiation risk counseling and provided written informed consent. The ALARA principle was strictly followed.

## 5. Conclusion

During MSE expansion, the buccal inclination of the maxillary first molar results from a combination of buccal expansion forces and coronal plane rotation of the ZMC. Optimal outcomes can be achieved by implementing age-appropriate MSE expansion protocols, strategically placing mini-implants, and minimizing molar band stress to mitigate dental effects such as molar buccal inclination and palatal cusp drop during arch expansion. Additionally, age-dependent midpalatal suture maturity plays a critical role in determining both skeletal and dental responses.

## Figures and Tables

**Table 1:** Angular measurement of zygomaticomaxillary complex, alveolar bone, and dental.

Angle	Unit	T1			T2			Treatment change		
		Mean	SD		Mean	SD		Mean	SD	*p*-Value
FZA	°	84.375	2.524		85.572	2.497		1.197	0.853	≤0.001*⁣*^*∗*^
FAA	°	70.859	2.254		72.127	2.460		1.195	1.320	≤0.001*⁣*^*∗*^
FDA	°	69.496	1.831		71.146	1.868		1.615	0.901	≤0.001*⁣*^*∗*^
ZMCA	°	95.898	5.438		98.039	5.023		2.036	1.644	≤0.001*⁣*^*∗*^
MFMA	°	10.713	6.351		15.063	6.138		4.315	3.195	≤0.001*⁣*^*∗*^
MSMA	°	15.885	7.368		18.198	7.367		2.313	1.800	≤0.001*⁣*^*∗*^

*Note:* Significant at *p＜*0.05.

Abbreviation: SD, standard deviation.

*⁣* ^*∗*^ *p＜*0.05.

**Table 2:** Comparison between angular change values.

Angular change 1	Angular change 2	MD	95% CI	*p*-Value
ΔFZA	ΔFAA	−0.023	−0.350–0.345	0.990
	ΔFDA	0.418	0.070–0.765	0.019*⁣*^*∗*^
ΔZMCA	ΔMFMA	−4.350	−6.501–−2.199	≤0.001*⁣*^*∗*^
	ΔMSMA	0.279	−0.932–0.374	0.399

*Note:* Significant at *p＜*0.05.

Abbreviations: CI, confidence interval; MD, mean difference.

*⁣* ^*∗*^ *p＜*0.05.

**Table 3:** Comparison of the proportion of zygomaticomaxillary complex (ZMC) rotation among age groups.

Age	Mean	95% CI	MD	95% CI	*p*-Value
≤18	0.712	0.668–0.765	0.201	0.137–0.266	≤0.001*⁣*^*∗*^
>18	0.511	0.463–0.558			

*Note:* Significant at *p＜*0.05.

Abbreviations: CI, confidence interval; MD, mean difference.

*⁣* ^*∗*^ *p＜*0.05.

## Data Availability

The data that support the findings of this study are available on request from the corresponding author. The data are not publicly available due to privacy or ethical restrictions.

## Nomenclature

MTD: Maxillary transverse deficiency
MARPE: Microscrew assisted rapid palatal expansion
MSE: Maxillary skeletal expander
RPE: Rapid palatal expansion
CBCT: Cone-beam computed tomography
ZMC: Zygomaxillary complex
FZA: Frontozygomatic angle
FAA: Frontoalveolar angle
FDA: Frontodental angle
ZMCA: Zygomaticomaxillary complex angle
MFMA: Maxillary first molar angle
MSMA: Maxillary second molar angle.
